# Effects of dexmedetomidine combined with intravenous general anesthesia on hemodynamics and inflammatory factors in patients undergoing laparoscopic colorectal cancer surgery

**DOI:** 10.20452/wiitm.2024.17891

**Published:** 2024-07-31

**Authors:** Chunling Liu, Yong Gui, Min Zeng, Zhidong Zhou

**Affiliations:** Department of Anesthesiology, Jiangxi Xinyu Traditional Chinese Medicine Hospital, Xinyu, Jiangxi Province, China; Department of Clinical Laboratory, Jiangxi Province Xinyu Traditional Chinese Medicine Hospital, Xinyu, Jiangxi Province, China; Department of Anesthesiology, Second Affiliated Hospital of Nanchang University, Nanchang, Jiangxi Province, China

**Keywords:** colorectal cancer, dexmedetomidine, hemodynamics, inflammatory factors, laparoscopic surgery

## Abstract

**INTRODUCTION:**

Surgery is the principal treatment option for early colorectal cancer (CRC). Anesthesia plays a crucial role in any surgery as it allows for a painless procedure. Dexmedetomidine is a local anesthetic that reduces pain and discomfort during surgery.

**AIM:**

The aim of this study was to investigate the efficacy of application of dexmedetomidine combined with total intravenous anesthesia in laparoscopic CRC surgery, with particular focus on its effects on patient hemodynamics and inflammatory factors.

**MATERIALS AND METHODS:**

For the purposes of this study, 80 patients undergoing elective laparoscopic rectal cancer surgery were selected and subsequently divided into 2 groups: the experimental group (0.5 µg/kg dexmedetomidine infused at a constant speed for 20 minutes, followed by 0.4 µg/kg/h dexmedetomidine) and the control group (0.5 µg/kg sufentanil infused at a constant speed for 20 minutes, followed by 0.4 µg/kg/h sufentanil). Each group comprised 40 patients. Hemodynamic parameters were recorded 1 minute before pumping dexmedetomidine or sufentanil (S0), 1 minute after pneumoperitoneum position (S1), 1 hour after pneumoperitoneum (S2), and 1 minute after elimination of air from the patient’s peritoneal cavity and position change (S3).

**RESULT:**

Systolic blood pressure (SBP) and diastolic blood pressure (DBP) at S1 and S2 in the experimental group were lower than in the control group (P <0.05). Heart rate (HR) and mean arterial pressure (MAP) at S1, S2, and S3 in the experimental group were lower, as compared with the control group (P <0.05). The levels of serum inflammatory factors (tumor necrosis factor α [TNF‑α], interleukin [IL]‑8, and IL‑6) and stress response indicators (plasma epinephrine, norepinephrine, and plasma cortisol) at S1, S2, and S3 in the experimental group were lower than in the control group (P <0.05). The expressions of TNF‑α, IL‑8, and IL‑6 in the experimental group negatively correlated with SBP and DBP (P <0.05), and with MAP and HR (P <0.001).

**CONCLUSION:**

Dexmedetomidine can effectively maintain hemodynamic stability and inhibit inflammatory and stress responses in patients undergoing laparoscopic CRC surgery, with its effect being superior to those of sufentanil.

## INTRODUCTION 

Colorectal cancer (CRC) is one of the most commonly diagnosed and highly prevalent malignancies globally. In recent years, its incidence and mortality rates have shown an upward trend, posing a significant public health concern.^1^^;^^2^ According to the data of the World Health Organization (WHO) and the International Agency for Research on Cancer (IARC), CRC ranks third among global causes of cancer‑related deaths, with varying degrees of incidence increases observed in both developed and developing countries.^3^ This trend underscores the importance of enhancing CRC diagnosis and treatment standards, as well as optimizing surgical strategies. Laparoscopic surgery has gradually become the primary approach for CRC surgery due to its advantages, such as minimal invasiveness, quick recovery, and reduced rate of complications.^4^ However, increased intra‑abdominal pressure and surgical manipulations during laparoscopic procedures can significantly impact the patient’s hemodynamic status. Furthermore, surgical trauma induces systemic stress responses, leading to elevated levels of inflammatory factors, such as interleukin 6 (IL‑6) and tumor necrosis factor α (TNF‑α), which, in turn, may increase the risk of postoperative complications and prolong recovery time.^5^^;^^6^^;^^7^^;^^8^ Therefore, effective handling of hemodynamic and inflammatory responses during surgery has become a critical focus in clinical anesthesia and perioperative management.

Dexmedetomidine is a novel and highly selective α2‑adrenergic receptor agonist that has been widely utilized in clinical anesthesia and intensive care settings in recent years.^9^^;^^10^ Apart from its excellent sedative, analgesic, and anxiolytic effects, dexmedetomidine significantly attenuates stress and inflammatory responses in patients. Its unique pharmacologic mechanisms contribute to maintaining hemodynamic stability, reducing the need for anesthetic agents, and improving postoperative cognitive function.^11^ Studies have demonstrated its significant benefits in high‑risk surgeries, such as cardiac and neurosurgical procedures, where it has proven to reduce the rate of intra‑and postoperative complications.^12^ Su et al^13^ analyzed the impact of dexmedetomidine on elderly patients treated at an intensive care unit following noncardiac surgery. The authors suggested that prophylactic low‑dose dexmedetomidine markedly reduced the incidence of delirium within the first postoperative week, with a favorable safety profile. In a randomized, placebo‑controlled trial performed across 6 academic hospitals in the United States, Turan et al^14^ found that initiating dexmedetomidine infusion at anesthesia induction and continuing it for 24 hours did not reduce the risk for postoperative atrial fibrillation or delirium in cardiac surgery patients. The application value of dexmedetomidine during the perioperative period warrants further evaluation, particularly in conjunction with total intravenous anesthesia (TIVA), a widely used anesthesia method for laparoscopic surgeries, characterized by stable anesthesia depth, rapid recovery, and minimal adverse effects, as compared with traditional inhalation anesthesia.^15^^;^^16^^;^^17^ Incorporating dexmedetomidine into TIVA can optimize anesthesia management, enhance surgical safety, and improve patient outcomes thanks to its unique pharmacologic actions.^18^ However, current research on the effects of dexmedetomidine combined with TIVA on hemodynamics and inflammatory factors in patients undergoing laparoscopic CRC surgery remains limited due to a lack of comprehensive clinical data support.

Building upon the aforementioned background, this study aimed to investigate the efficacy of application of dexmedetomidine combined with TIVA in laparoscopic CRC surgery, with particular focus on its effects on patient hemodynamics and inflammatory factors. The study employed a randomized controlled design to compare the dexmedetomidine group with a control group, assessing intraoperative hemodynamic parameters, postoperative inflammatory cytokine levels, and recovery outcomes. It is anticipated that this study can further validate the safety and effectiveness of dexmedetomidine in laparoscopic CRC surgery, providing scientific evidence for clinical anesthesia practice and offering new insights and approaches to perioperative management of CRC surgery patients.

## AIM 

The aim of this study was to evaluate the effects of dexmedetomidine combined with intravenous general anesthesia on hemodynamics and inflammatory factors in patients undergoing laparoscopic CRC surgery.

## MATERIALS AND METHODS 

### Study participants 

From September 1, 2019 to February 28, 2023, a total of 80 patients undergoing elective laparoscopic rectal cancer surgery at the Jiangxi Xinyu Traditional Chinese Medicine Hospital were selected. The sample included 33 men and 47 women, aged 40 to 72 years. Their body weight ranged from 47 to 73 kg.

The participants agreed to sign informed consent forms, including the consent of their family members. The Hospital Ethics Society approved the study protocol (2019004).

Inclusion criteria were as follows: 1) primary CRC confirmed by pathology, 2) eligibility for surgical treatment, 3) no concomitant autoimmune disease, 4) no infection within 1 month before surgery, and 5) complete clinical data.

Exclusion criteria comprised: 1) a history of treatment with nonsteroidal anti‑inflammatory drugs, 2) arrhythmia, 3) long‑term use of sedative drugs before surgery, 4) abnormal liver and kidney function, 5) excessive drinking, and 6) obesity.

### Anesthesia methods 

The patients were divided into 2 groups, each comprising 40 individuals. Those assigned to the experimental group were administered 0.5 µg/kg of dexmedetomidine at a constant speed for 20 minutes, followed by 0.4 µg/kg/h of dexmedetomidine. The control patients were given 0.5 µg/kg of sufentanil for 20 minutes, followed by a continuous infusion of 0.4 µg/kg/h of sufentanil.

The patients did not receive any preanesthetic medication prior to entering the operating room, and once there, their peripheral antecubital vein was opened. Before the induction of anesthesia, the patients were given lactated Ringer solution (Qingdao Jisskang Biotechnology Co., Ltd., Qingdao, China) at a dose of 8 ml/kg body weight. Physiological parameters, such as cardiac electrical activity, body temperature, blood pressure, and end‑tidal carbon dioxide were displayed on a multiparameter monitor YH‑JHY‑800A (Sichuan Keyicheng Technology Co., Ltd., Chengdu, China). The FloTrac sensor (Shanghai Zhengxi Medical Device Co., Ltd., Shanghai, China) was used to evaluate the patients’ hemodynamics, and a bispectral index sensor was employed to monitor the depth of anesthesia. After 10 minutes, blood samples were collected to determine baseline indicator values, and then the patients in the experimental and control groups were given dexmedetomidine and sufentanil, respectively.

The anesthesia in both groups was administered via the same intravenous method. Anesthesia induction drugs included midazolam at 0.04 mg/kg body weight, fentanyl at 4 μg/kg body weight, Esmeron at 0.6 mg/kg body weight, and propofol at 1.5 mg/kg body weight. The anesthesia was maintained during surgery with the inhalation of a sevoflurane and remifentanil infusion (at 0.1 μg/kg per minute) through an intravenous pump. Cisatracurium was added as needed to maintain muscle relaxation. Meanwhile, the concentration of sevoflurane was adjusted to the bispectral index to keep the bispectral index value between 50 and 55. After the surgery, patients’ respiratory parameters were regulated to maintain the end‑tidal carbon dioxide partial pressure (PetCO_2 _) within the range of 35–45 mm Hg (1 mm Hg = 0.133 kPa). If the bispectral index value was within the normal range but the intraoperative systolic blood pressure (SBP) value exceeded 160 mm Hg or was 20% above the preoperative basal level, 50–100 μg of nitroglycerin was administered to manage the hypertensive condition. If SBP fell below 90 mm Hg or 20% below the preoperative basal level, 3–6 mg of ephedrine was given to manage hypotension. When heart rate (HR) exceeded 100 bpm, 20 mg of esmolol was administered. When the air in the abdominal cavity was eliminated, the patient was moved from a supine position to a different one, and the infusion of dexmedetomidine was stopped. Fentanyl (at 2 μg/kg body weight) was administered intravenously and an intravenous pump was connected for analgesia. Other maintenance medications were administered until the end of the procedure. All surgeries were performed by the same surgeon and the same anesthesiologist in the same operating room. All of them were scheduled as the first procedures in the morning.

### Observation indicators 

Respiratory rate, pulse oxygen saturation, PetCO _2 _, cardiac output (CO), and bispectral index were recorded.

Hemodynamic parameters were recorded 1 minute before pumping dexmedetomidine or sufentanil (S0), 1 minute after pneumoperitoneum position (S1), 1 hour after pneumoperitoneum (S2), and 1 minute after elimination of air from the patient’s peritoneal cavity and position change (S3). They included SBP, diastolic blood pressure (DBP), HR, mean arterial pressure (MAP), CO, and cardiac index (CI).

A sample containing 5 ml of radial arterial blood was collected from the patients at S0, S1, S2, and S3 to measure the levels of glucose, plasma epinephrine, norepinephrine, plasma cortisol, and inflammatory factors (TNF‑α, IL‑8, IL‑6).

The frequency of ephedrine, nitroglycerin, atropine, and esmolol administration during the perioperative period was also recorded.

### Sample size calculation method 

The required number of patients was calculated according to the equation

\displaystyle n = 2 \times \frac{\left( \sigma \times \frac{Z_{\alpha}}{2} + \sigma \times Z_{\beta} \right)^2}{\sigma^2}

where n was the sample size and σ represented the standard deviation. The bilateral significance was set at α = 0.05, the assurance degree, at 80%, and the statistical power, at β = 0.85.

### Statistical analysis 

For data analysis, SPSS 19.0 statistical software was used (IBM Corp., Armonk, New York, United States). Measurement data were expressed as mean (SD), and count data were expressed as percentage. The repeated measures analysis of variance (ANOVA) was adopted for comparisons between the groups, and the 2‑way ANOVA was adopted for comparisons within a single group. A P value below 0.05 was considered significant for a 2‑sided test.

## RESULTS 

### Comparison of general patient data 

As illustrated inFigure 1 , the mean (SD) age of the patients in the experimental group was 65 (6) years, their mean (SD) height was 160 (10) cm, their mean (SD) weight was 59 (3) kg, the mean (SD) operation time was 165.45 (13.36) minutes, and the mean (SD) intraoperative fluid infusion volume was 1711.03 (122.95) ml. The group comprised 17 men and 23 women. The mean (SD) age of the control participants was 66 (8) years, their mean (SD) height was 158 (7) cm, their mean (SD) weight was 61 (6) kg, the mean (SD) operation time was 170.72 (11.95) minutes, and the mean (SD) intraoperative fluid infusion volume was 1679.54 (108.34) ml. There were 16 men and 24 women in the control group. There was no significant difference in age, height, weight, operation time, intraoperative fluid infusion volume, or sex distribution between the 2 groups.

**Figure 1:**
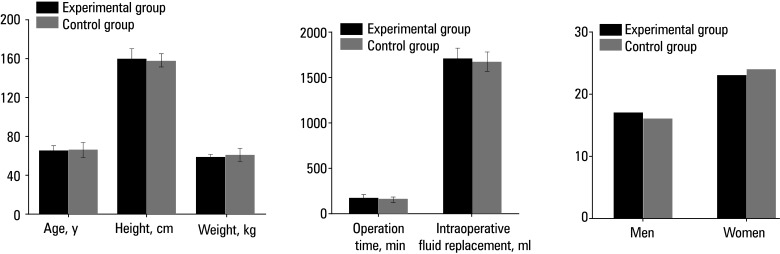
Comparison of general patient data; **A** – age, height, weight; B – operation time and volume of intraoperative fluid infusion; **C** – sex

**Figure 2:**
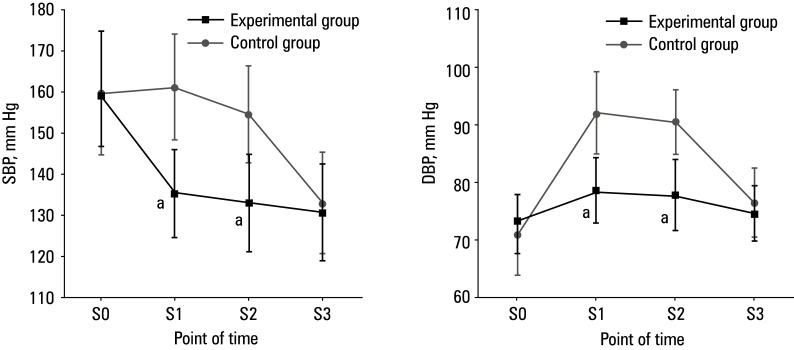
Comparison of hemodynamic parameters; **A** – systolic blood pressure (SBP);** B** – diastolic blood pressure (DBP) **a** *P* <0.05 for experimental vs control group

Abbreviations: S0, evaluation performed 1 minute before pumping dexmedetomidine or sufentanil; S1, evaluation

performed 1 minute after pneumoperitoneum position; S2, evaluation performed 1 hour after pneumoperitoneum;

S3, evaluation performed 1 minute after elimination of air from the patient’s peritoneal cavity and position change

**Figure 3:**
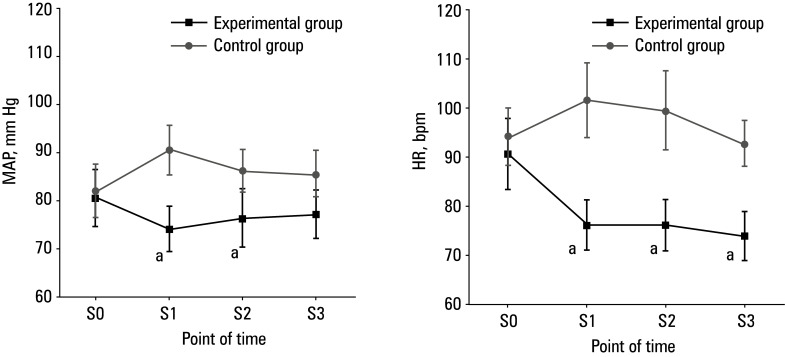
Comparison of hemodynamic parameters; **A **– mean arterial blood pressure (MAP); **B** – heart rate (HR)

**a ***P <⁠*0.05 for experimental vs control group

Abbreviations: see Figure 2

### Comparison of hemodynamic parameters 

As presented in Figure 2, SBP and DBP were similar in both groups at S0 and S3. SBP and DBP in the experimental group were lower than in the control group at S1 and S2 (P <0.05).

**Figure 4:**
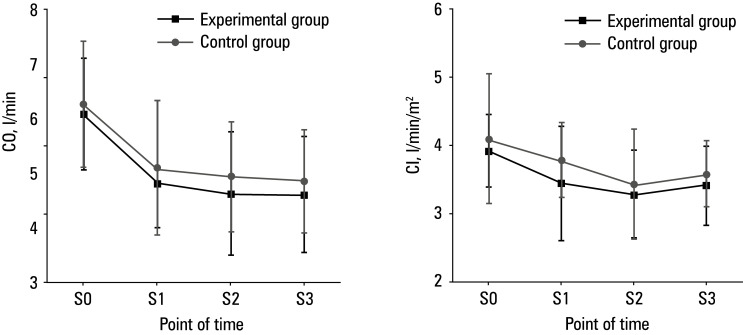
Comparison of hemodynamic parameters between the groups; **A** – cardiac output (CO); **B** – cardiac index (CI)

Abbreviations: seeFigure 2

With respect to hemodynamic parameters, HR and MAP were similar between the 2 groups at S0, whereas they were lower in the experimental group than in the control group at S1, S2, and S3 (P <0.05)Figure 3. CO and CI did not differ between the 2 groups at any of the evaluated time points Figure 4.

**Figure 5:**
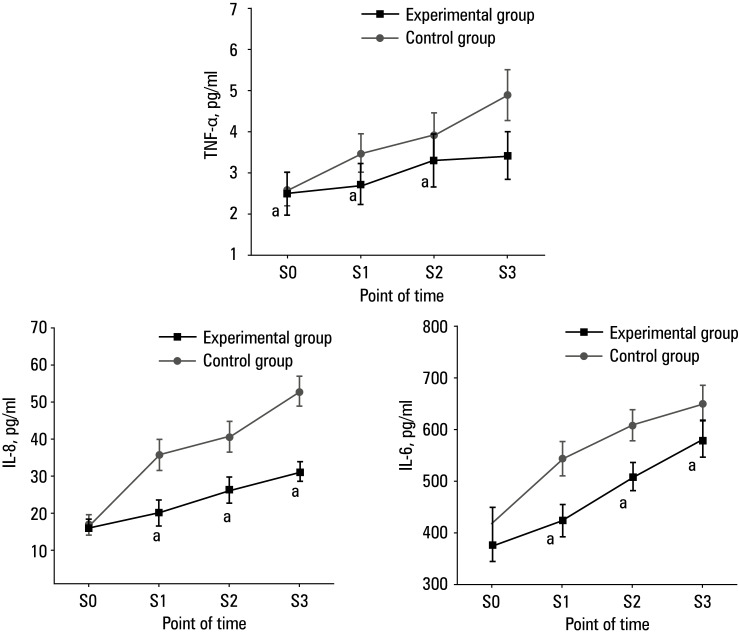
Comparison of serum inflammatory factor levels; **A** – tumor necrosis factor α (TNF-α); **B** – interleukin 8 (IL-8), **C** – interleukin 6 (IL-6)

**a ***P <⁠*0.05 for experimental vs control group

Abbreviations: seeFigure 2

### Comparison of serum inflammatory factor levels 

Serum levels of the assessed inflammatory factors (TNF‑α, IL‑8, and IL‑6) were similar between both groups at S0, whereas at S1, S2, and S3, they were lower in the experimental group, as compared with the control group (P <0.05)Figure 5.

### Comparison of stress response indicators 

**Figure 6:**
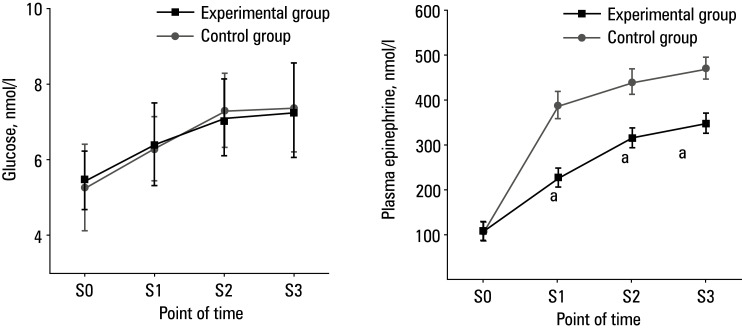
Comparison of stress response indicator levels; **A** – blood glucose; **B** – plasma epinephrine

As shown in Figure 6, glucose and plasma epinephrine levels in both groups were comparable at S0. There was no obvious difference in the blood glucose level at S1, S2, and S3 between the 2 groups. The plasma epinephrine level in the experimental group was lower than in the control group at S1, S2, and S3 (P <0.05).

**Figure 7:**
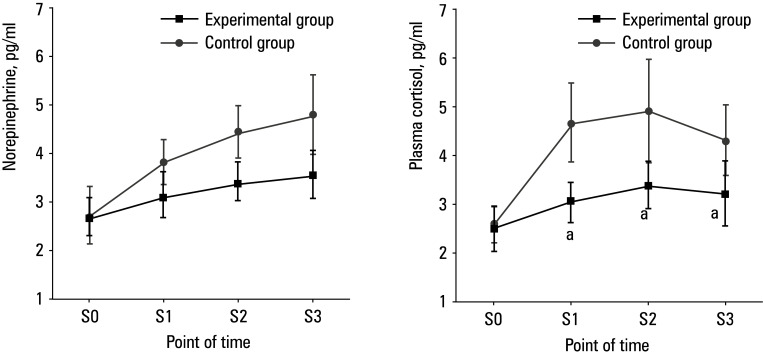
Comparison of stress response indicator levels; **A** – norepinephrine; **B** – plasma cortisol

There was no difference in the levels of stress response indicators, norepinephrine and plasma cortisol, between the 2 groups at S0. However, these indices were lower in the experimental group than in the control group at S1, S2, and S3 (P <0.05)Figure 7.

### Correlation analysis of serum inflammatory factors and hemodynamic parameters in the experimental group 

According to the Pearson correlation analysis, there was a significant negative correlation between serum inflammatory factor levels (TNF‑α, IL‑8, and IL‑6) and some of the hemodynamic parameters in the experimental group. Specifically, TNF‑α, IL‑8, and IL‑6 levels were significantly negatively associated with SBP and DBP values. Moreover, there were strong negative correlations (P <0.001) between the levels of these inflammatory factors and MAP and HR. These findings suggest a strong association between elevated levels of inflammatory markers and decreased hemodynamic stability in patients undergoing experimental procedures Table 1 ;Table 2 ;Table 3.

**a ***P <⁠*0.05 for experimental vs control group

Abbreviations: see Figure 2

**a ***P <⁠*0.05 for experimental vs control group

Abbreviations: see Figure 2

## DISCUSSION 

Anesthesia management in rectal cancer surgery is crucial and typically involves propofol or etomidate as general anesthesia induction agents, with sevoflurane or desflurane used to maintain anesthesia depth and hemodynamic stability. Specifically, dexmedetomidine plays a significant role in intraoperative pain management due to its ultrashort‑acting profile. This characteristic allows for rapid adjustment of analgesic effects and helps maintain hemodynamic stability when surgical stimulation is reduced.^19^^;^^20^^;^^21^ Therefore, we evaluated 80 patients undergoing elective laparoscopic rectal cancer surgery who were randomized to receive either dexmedetomidine or sufentanil combined with TIVA. Clinical data of the 2 groups were compared. There were similarities in age, height, weight, and sex distribution of patients, as well as in operation time and intraoperative fluid infusion volume. This result indicates that at the beginning of the study, the baseline characteristics of the 2 patient groups were largely balanced, which is advantageous for subsequent comparisons of surgical and other treatment effects on outcomes.

We found that certain hemodynamic parameters (SBP and DBP) were significantly inferior at S1 and S2 in the experimental group, as compared with the control group, which is in line with the results obtained by Hu et al.^22^ Our findings indicated that dexmedetomidine was more effective than sufentanil in maintaining stable BP, thereby preventing SBP and DBP fluctuations. This provides valuable insights for clinical practice and research, contributing to the optimization of anesthesia and analgesic strategies to enhance surgical safety and postoperative outcomes for patients. 

By monitoring HR and MAP, doctors can assess the functional status of the cardiovascular system, as abnormal HR and MAP may be related to cardiovascular diseases, metabolic disorders, drug reactions, or other health problems.^23^ In the experimental group, HR and MAP at S1, S2, and S3 were significantly lower than in the control group, which shows that dexmedetomidine could effectively maintain patient hemodynamic stability, and its intervention effect was better than that of sufentanil.

**Table 1:** Correlation between tumor necrosis factor α level and hemodynamic parameters

Correlation indicator	SBP	DBP	MAP	HR
r	–0.258	–0.291	–0.466	–0.484
*P *value	0.007	0.015	0	0

Abbreviations: see Figure 2 and Figure 3

**Table 2:** Correlation between interleukin 8 level and hemodynamic parameters

Correlation indicator	SBP	DBP	MAP	HR
r	–0.305	–0.177	–0.389	–0.475
*P *value	0.003	0.016	0	0

Abbreviations: see Figure 2 and Figure 3

**Table 3:** Correlation between interleukin 6 level and hemodynamic parameters

Correlation indicator	SBP	DBP	MAP	HR
r	–0.127	–0.143	–0.504	–0.387
*P *value	0.018	0.022	0	0

Abbreviations: see Figure 2 and Figure 3

In addition, serum levels of TNF‑α, IL‑8, and IL‑6 in the experimental group were significantly lower than in the control group at S1, S2, and S3. Inflammatory factors refer to a class of molecules that are produced in the process of inflammation and play an important role in the regulation of the immune system and inflammatory response. Changes in their levels can reflect the severity of the inflammatory response. In this respect, we showed that dexmedetomidine can effectively inhibit patients’ inflammatory response and help avoid tissue damage.^24^ Perioperative stress response in patients can lead to various adverse outcomes, including increased risk of surgical complications, delayed postoperative recovery, heightened pain perception, immune suppression, and psychological effects. Timely and effective management of these stress responses is crucial for reducing complication risks, promoting postoperative recovery, and improving overall surgical success rates.^25^ We found that the levels of stress response indicators (plasma epinephrine, norepinephrine, and plasma cortisol) were significantly lower in the experimental group, as compared with the control group, at S1, S2, and S3. This finding indicates that dexmedetomidine could effectively inhibit the stress response of patients undergoing laparoscopic CRC surgery, thereby alleviating immunosuppression and improving postoperative recovery. This study further utilized Pearson correlation analysis and found that in the experimental group, the expression levels of TNF‑α, IL‑8, and IL‑6 negatively correlated with hemodynamic parameters, SBP and DBP (P <0.05), and strongly negatively correlated with MAP and HR (P <0.001). This suggests that, as indicators of stress response, TNF‑α, IL‑8, and other inflammatory markers may adversely affect hemodynamic parameters through certain mechanisms. These findings highlight the importance of close monitoring and management of inflammatory and stress responses during surgery to improve clinical outcomes of patients.

## CONCLUSIONS 

We showed that dexmedetomidine could effectively maintain hemodynamic stability and inhibit inflammatory and stress responses in patients undergoing laparoscopic CRC surgery, and that its effect was superior to that of sufentanil. However, it should be noted that the sample size was relatively small. Also, the number of assessed indicators was limited, and these indicators could be affected by a variety of internal and external factors, potentially leading to bias. Therefore, future studies should comprise more patients from a wider variety of sources for more indepth findings. In conclusion, the results provided a reference for perioperative anesthesia in laparoscopic rectal cancer surgery.

## References

[BIBR-1] Liang Z., Zeng G.X., Wan W. (2023). The unique genetic mutation characteristics based on large panel next‐generation sequencing (NGS) detection in multiple primary lung cancers (MPLC) patients. Discov Med.

[BIBR-2] Huang X.P., Chen J.J., Xiang H.Q., Yu X.T. (2022). Maslinic acid suppresses cervical cancer growth by inducing apoptosis through a P53‐dependent and bcl related pathway in vitro. J Biol Regulat Homeost Agent.

[BIBR-3] Cao M., Feng R. (2023). Application of refined management under the guidance of temperature agitation monitoring in recovery period management of colorectal cancer patients undergoing laparoscopic surgery under general anesthesia. Acta Medica Mediterr.

[BIBR-4] Liu H. (2022). Pan‐cancer profiles of the cuproptosis gene set. Am J Cancer Res.

[BIBR-5] Baidoun F., Elshiwy K., Elkeraie Y. (2021). Colorectal cancer epidemiology: recent trends and impact on outcomes. Curr Drug Targets.

[BIBR-6] Heinimann K. (2018). Hereditary colorectal cancer: clinics, diagnostics and management [in German. Ther Umsch.

[BIBR-7] Eng C., Jácome A.A., Agarwal R. (2022). A comprehensive framework for early‐onset colorectal cancer research. Lancet Oncol.

[BIBR-8] Zielińska A., Włodarczyk M., Makaro A. (2021). Management of pain in colorectal cancer patients. Crit Rev Oncol Hematol.

[BIBR-9] Jin K., Ren C., Liu Y. (2020). An update on colorectal cancer microenvironment, epigenetic and immunotherapy. Int Immunopharmacol.

[BIBR-10] Zhou E., Rifkin S. (2021). Colorectal cancer and diet: risk versus prevention, is diet an intervention?. Gastroenterol Clin North Am.

[BIBR-11] Drogan C., Kupfer S.S. (2022). Colorectal cancer screening recommendations and outcomes in lynch syndrome. Gastrointest Endosc Clin N Am.

[BIBR-12] Ahmad R., Singh J.K., Wunnava A. (2021). Emerging trends in colorectal cancer: dysregulated signaling pathways (review. Int J Mol Med.

[BIBR-13] Su X., Meng Z.T., Wu X.H. (2016). Dexmedetomidine for prevention of delirium in elderly patients after non‐cardiac surgery: a randomised, double‐blind, placebo‐controlled trial. Lancet.

[BIBR-14] Turan A., Duncan A., Leung S. (2020). Dexmedetomidine for reduction of atrial fibrillation and delirium after cardiac surgery (DECADE): a randomised placebo‐controlled trial. Lancet.

[BIBR-15] Sun W., Li F., Wang X. (2021). Effects of dexmedetomidine on patients undergoing laparoscopic surgery for colorectal cancer. J Surg Res.

[BIBR-16] Ford D.J., Dingwall A.K. (2015). The cancer COMPASS: navigating the functions of MLL complexes in cancer. Cancer Genet.

[BIBR-17] Li Y., Liu H. (2022). Clinical powers of aminoacyl tRNA synthetase complex interacting multifunctional protein 1 (AIMP1) for head‐neck squamous cell carcinoma. Cancer Biomark.

[BIBR-18] Zhang J., Liu G., Zhang F. (2019). Analysis of postoperative cognitive dysfunction and influencing factors of dexmedetomidine anesthesia in elderly patients with colorectal cancer. Oncol Lett.

[BIBR-19] Zhang Z., Hao D. (2023). Effect of transversus abdominis plane block combined with low‐dose dexmedetomidine on elderly patients undergoing laparoscopic colectomy. Wideochir Inne Tech Maloinwazyjne.

[BIBR-20] Chen C., Huang P., Lai L. (2016). Dexmedetomidine improves gastrointestinal motility after laparoscopic resection of colorectal cancer: a randomized clinical trial. Medicine (Baltimore.

[BIBR-21] Fares K.M., Mohamed S.A., Abd El‐Rahman A.M. (2015). Efficacy and safety of intraperitoneal dexmedetomidine with bupivacaine in laparoscopic colorectal cancer surgery, a randomized trial. Pain Med.

[BIBR-22] Hu J., Gong C., Xiao X. (2023). Association between intraoperative dexmedetomidine and all‐cause mortality and recurrence after laparoscopic resection of colorectal cancer: follow‐up analysis of a previous randomized controlled trial. Front Oncol.

[BIBR-23] Freeman J., Buggy D.J. (2018). Modelling the effects of perioperative interventions on cancer outcome: lessons from dexmedetomidine. Br J Anaesth.

[BIBR-24] An G., Wang G., Zhao B. (2022). Opioid‐free anesthesia compared to opioid anesthesia for laparoscopic radical colectomy with pain threshold index monitoring: a randomized controlled study. BMC Anesthesiol.

[BIBR-25] Kinugasa H., Higashi R., Miyahara K. (2018). Dexmedetomidine for conscious sedation with colorectal endoscopic submucosal dissection: a prospective double‐blind randomized controlled study. Clin Transl Gastroenterol.

